# Successful repair of transplant renal artery aneurysm (TRAA)

**DOI:** 10.1186/s12894-023-01280-z

**Published:** 2023-07-31

**Authors:** Collin Elijah Rui Hung Ho, Wei Zheng So, Julian Wong, Ho Yee Tiong

**Affiliations:** 1 Yong Loo Lin School of Medicine, National University of Singapore, Singapore, Singapore; 2 National University Hospital, Singapore, Singapore

**Keywords:** Repair, Transplant renal artery aneurysm, Saphenous vein

## Abstract

**Background:**

Transplant renal artery aneurysm (TRAA) is rare. TRAA that develops post transplantation consists of 0.10% of the vascular complications post renal transplant (Transplant Proc 41:1609-1614, 2009; Indian J Urol 29:42-47, 2013).

**Case presentation:**

We report a case of TRAA in an asymptomatic young female. CT angiogram with detailed 3D reconstruction showed a 2.6 × 2.2 cm wide neck saccular TRAA arising from the anterior segmental branch of the graft renal artery (Figs. 2 and 3). A multidisciplinary team of interventional radiologists, vascular and urologist was involved for preoperative surgical planning and unique repair methods. Endovascular and percutaneous approaches were deemed not feasible, and an open in vivo approach with a saphenous vein graft was taken.

**Conclusion:**

TRAA, albeit rare, is a complication that can occur post renal transplant. In-vivo surgical repair of TRAA is feasible with a multidisciplinary approach and careful preoperative planning. Saphenous vein graft is still a versatile graft and can be used as a conduit successfully.

## Introduction

Renal transplantation is the definitive treatment for end-stage renal failure. Vascular complications, albeit rare can potentially lead to allograft loss [1]. Anastomotic renal artery aneurysms are often iatrogenic. Transplant renal artery aneurysm (TRAA) when detected in renal grafts are often resected at the back-table before grafting. However, TRAA that develops de novo post transplantation poses unique challenge in the management.

## Case presentation

A 30-year-old lady underwent an uncomplicated living related renal transplant in 2006 at the age of 17. She initially presented with uremia and hypertensive urgency. She was diagnosed with End Stage Renal Disease (ESRD) and was subsequently started on hemodialysis in that same admission. She then received a Living Donor Kidney Transplant (LDKT) from her mother 4 months later. Post-transplant, she was asymptomatic, and her estimated glomerular filtration rate (eGFR) was 57 ml/min. She was a non-smoker with no other significant past medical history. There was no history of intravenous drug abuse or atherosclerosis of blood vessels.

**Fig. 1:**
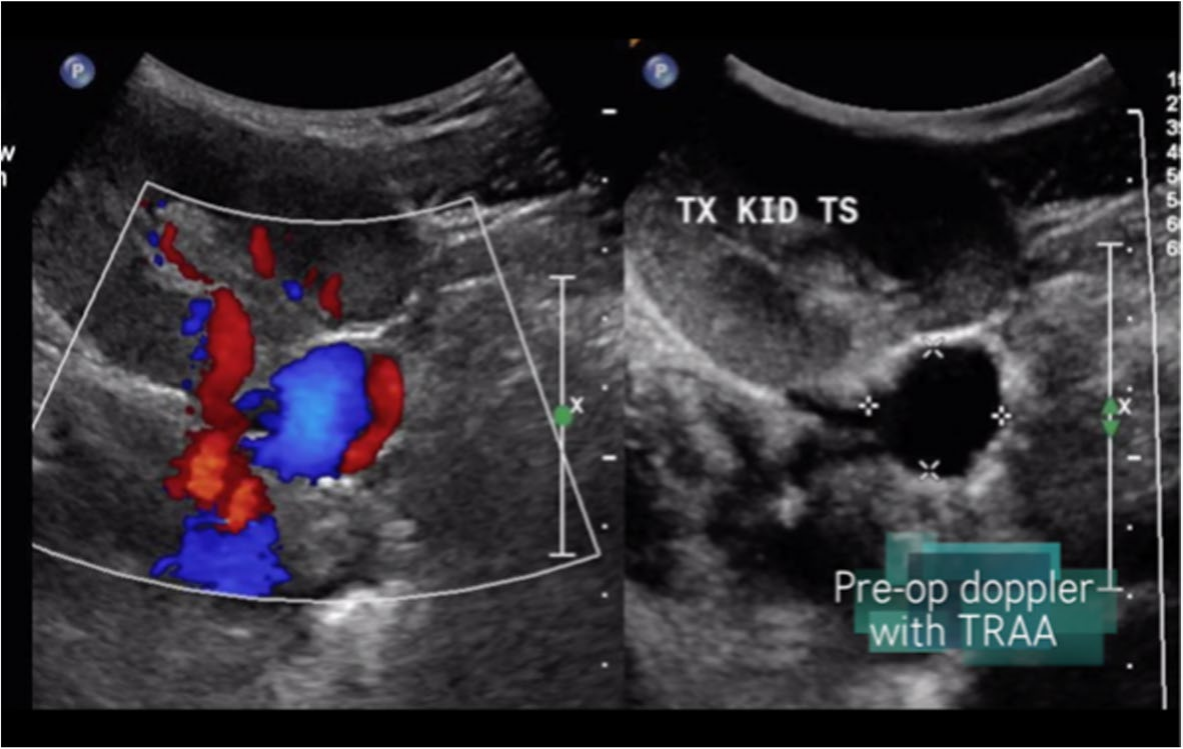
Ultrasound doppler showing TRAA

She was on regular follow up and during a routine follow up ultrasound in 2018, we incidentally detected a saccular TRAA (Fig. 1).

She was asymptomatic, and abdominal examination was unremarkable.

A timeline of her clinical course is as follows.

- March 2006 – Presented with uremia and hypertensive urgency (ESRD)
- July 2006 – LDKT from her mother

- 2018 – TRAA incidentally detected on Doppler Ultrasound Screening

## Diagnostic assessment

**Fig. 2:**
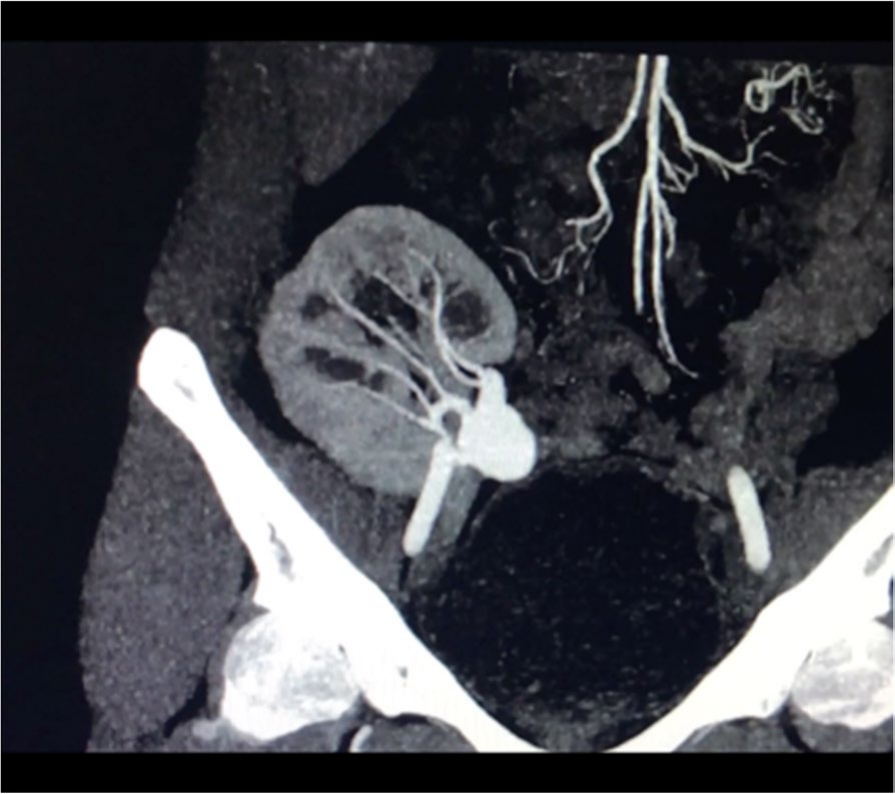
CT angiography showing large TRAA

Doppler ultrasound of the renal vessels yearly is routinely used for follow-up. At the time of transplantation, pre operative imaging did not detect any TRAA in the donor kidney. There were no kidney biopsies performed for cause until TRAA was detected. After suspicion of a TRAA on doppler ultrasound (Fig. 1), a multi-sliced computed tomography angiogram with 3D reconstruction is essential to assess TRAA anatomy and for surgical planning, as seen in our patient (Fig. 2).

**Fig. 3:**
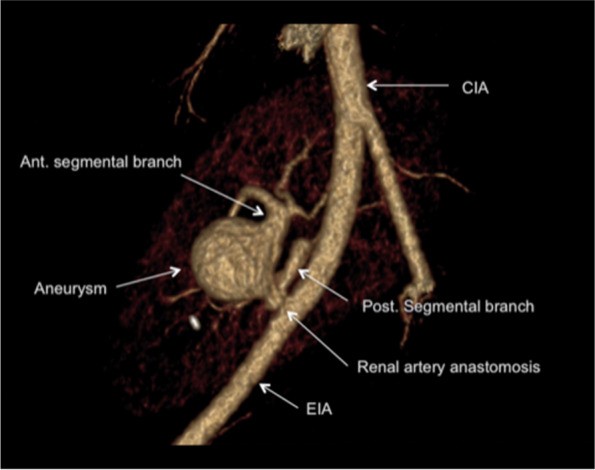
3D reconstruction image of the CT Angiogram showing TRAA arising from the anterior segmental branch

Computed Tomography (CT) angiogram with a detailed 3D reconstruction was then done, which showed a 2.6 × 2.2 cm wide neck saccular TRAA arising from the anterior segmental branch of the graft renal artery (Figs. 2 and 3). Intrarenal vasculature was noted to be normal.

Her eGFR was 57 ml/min before the repair of the TRAA.

## Therapeutic intervention

A multidisciplinary meeting was held with a radiologist, vascular surgeon and urologist and treatment options were discussed for TRAA including endovascular stenting, percutaneous thrombin injection and surgical repair. Endovascular stenting is often used in renal artery pseudoaneurysm and it requires adequate proximal and distal landing zone [2]. Endovascular approach was excluded due to the short take off of the renal artery and lack of proper landing zone (Fig. 3). With percutaneous approach deemed not feasible, the decision for open surgical approach with in vivo repair was made. Involving a multidisciplinary team is a key step in successful management of TRAA.

During the surgical planning, the saphenous vein was identified as an appropriate conduit. It is a commonly used autologous graft for various vascular reconstruction. Although there are some reports on delayed saphenous vein graft aneurysms [3], the saphenous vein’s caliber, length, ease of exploration and little venous drainage consequences when sacrificed has made it a versatile autologous graft [3].

A midline laparotomy was performed for direct access to the anteriorly placed aneurysm and iliac vessels. A midline transperitoneal approach provided direct access to the renal transplant graft hilum and TRAA. The Right common iliac artery (CIA) and right external iliac artery (EIA) were isolated for possible emergency clamping. We planned not to mobilize the allograft to reduce risk of injury to the graft.

**Fig. 4:**
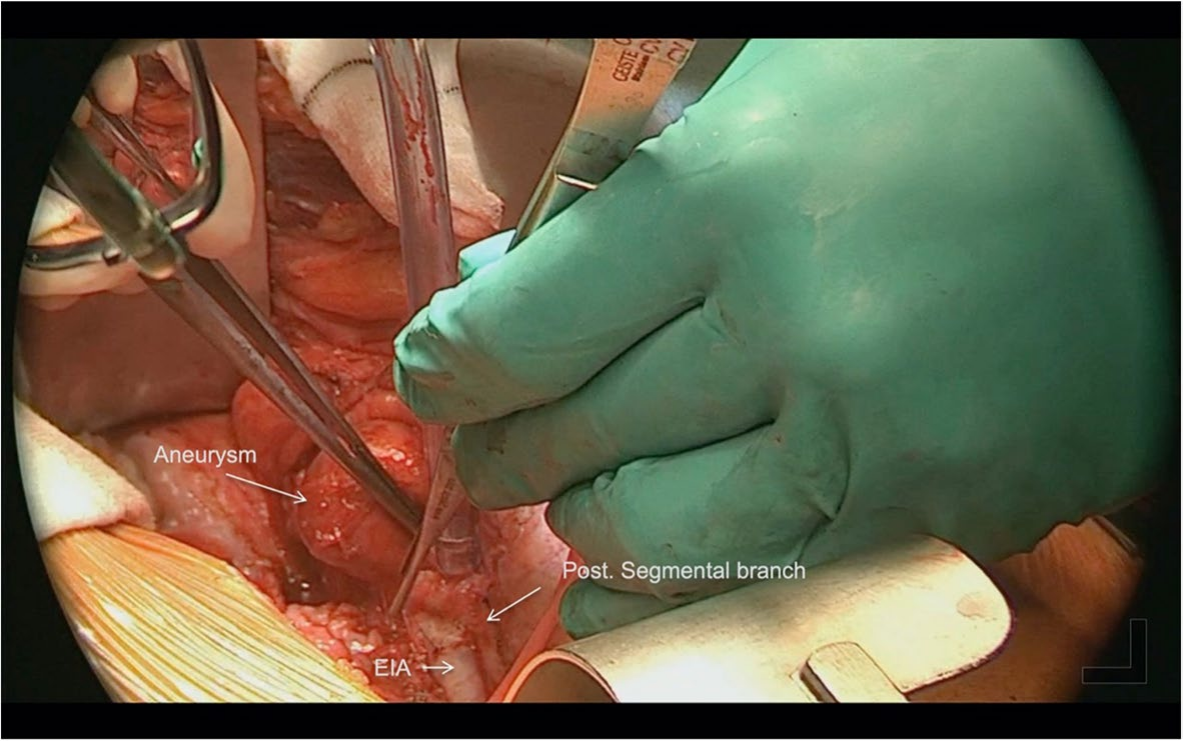
Intraoperative image showing renal artery aneurysm arising from anterior segmental branch, posterior segmental branch of renal artery and external iliac artery

TRAA was dissected down to the neck and the branches were carefully dissected to gain adequate length (Fig. 4). Renal vein was identified and isolated. Saphenous vein graft was harvested and prepared.

**Fig. 5:**
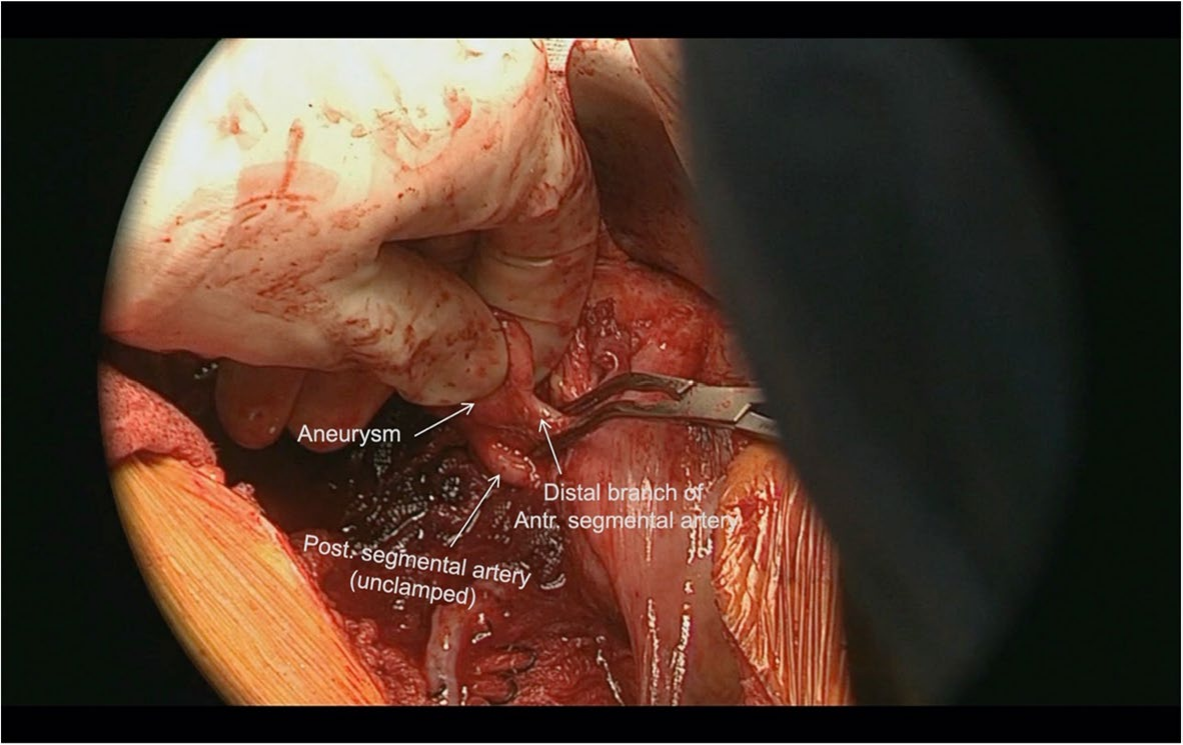
Anterior segmental branch is clamped distal to the aneurysm. Posterior segmental branch is left unclamped

Partial nephrectomy segmental clamping principles were followed to isolate the aneurysm. The proximal end of the anterior segmental artery was ligated and the distal branch of the anterior segmental artery was clamped. The posterior segmental artery and renal vein were left unclamped to ensure perfusion to the remaining kidney (Fig. 5).

**Fig. 6:**
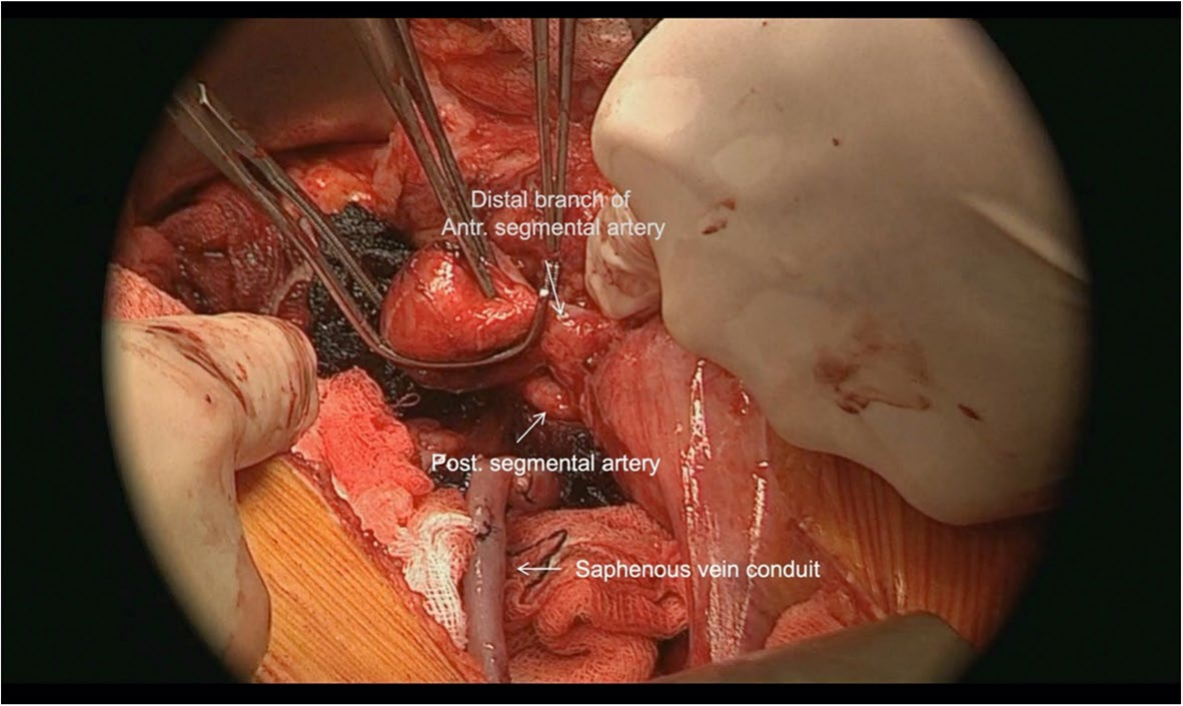
Saphenous vein graft is prepared and one end is anastomosed to common iliac artery

The proximal end of the saphenous vein was anastomosed to the common iliac artery (Fig. 6).

In-vivo excision of the TRAA was performed.

**Fig. 7:**
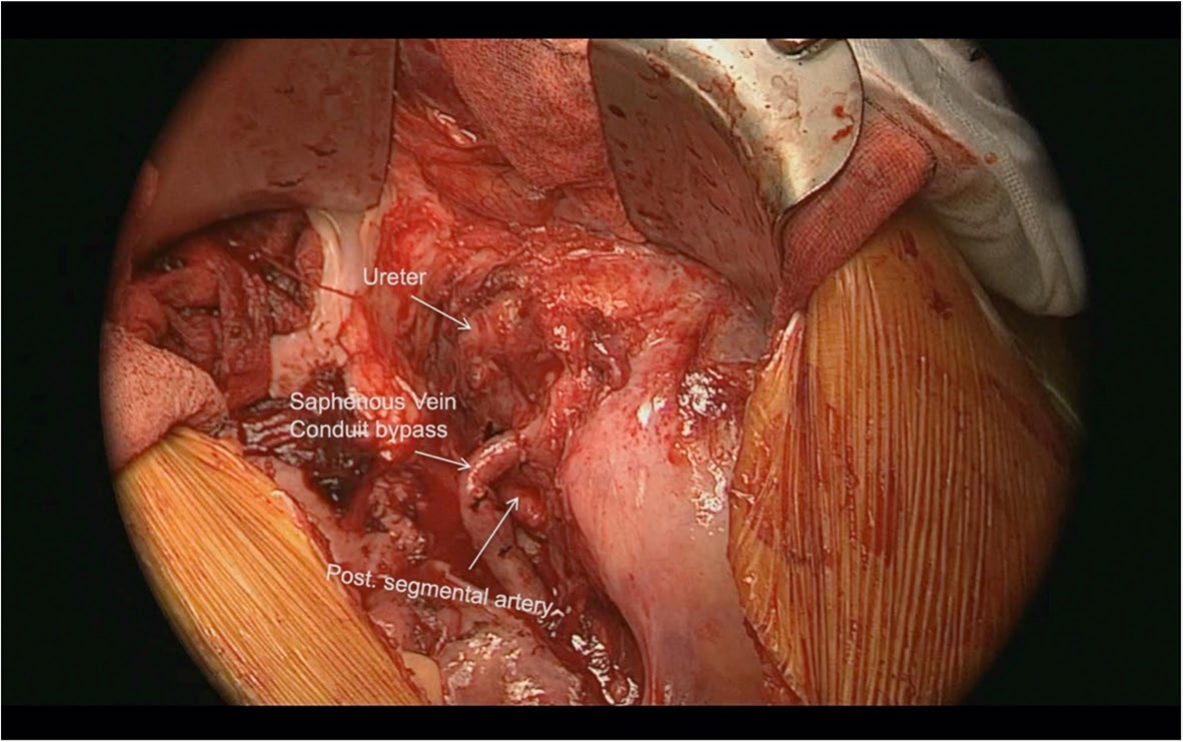
Distal end of the saphenous vein graft is anastomosed to the distal divided end of the anterior segmental artery

The saphenous vein graft was trimmed to the desired length, and anastomosed to the distal divided end of the anterior segmental branch of the renal artery. The saphenous vein was thus used as a bypass from the right CIA to the distal end of the anterior segmental branch of the renal artery (Fig. 7).

Hemostasis was secured followed by careful inspection of the anastomosis before closure.

The total operative time was 148 min, with an estimated blood loss of 500 ml. The warm ischemic time of the allograft was 20 min.

There was no clinical evidence of a mycotic aneurysm. Grocott methenamine silver stain and Ziehl–Neelsen stain were performed on the aneurysm and both stains were negative for fungal organisms and acid fast bacilli.

Since the repair, there has not been a recurrence. A CT Angiogram 6 months post TRAA Repair as well as the latest Doppler ultrasound in 2021 did not demonstrate new aneurysms.

Her renal function has been stable post TRAA repair. Her latest eGFR and Creatinine levels in 2023 are 54 ml/min and 114 µmol/L respectively.

There were no adverse and unanticipated events.

## Discussion

Vascular complications post renal transplant are not uncommon and may lead to allograft loss. Common vascular complications include Transplant Renal Artery Stenosis, Transplant Renal Artery Thrombosis. Extrarenal pseudoaneurysm formation rare with an estimated prevalence of < 1% [1, 4]. Pseudoaneurysms managed with endovascular stenting/surgical excision often results in higher rate of transplant nephrectomy [5].

True renal artery aneurysm after transplantation is rare and even rarer to present as a late complication during follow up [6]. TRAA are usually detected during early stages of follow up. Aneurysms are often seen as pseudoaneurysm arising from the anastomotic site. A true aneurysm contains all three layers of the blood vessel wall, unlike a pseudoaneurysm [7]. Most of TRAA are asymptomatic but some present with fever, symptoms arising from compression of nearby structures, hypertension, reduced allograft function and rarely life-threatening hemorrhage [8]. There is a potential risk of catastrophic complications if TRAA is left untreated, including aneurysm rupture, renal artery thrombosis and embolus causing renal infarction and dissections [9].

Various etiologies have been associated with aneurysm formation. Pseudoaneurysm is often due to poor surgical technique, suture rupture, vessel wall ischemia or mycotic infection [10].

True aneurysm is often idiopathic and association with immunological etiology is yet to be proven [8]. Indications for intervention reported in literature are symptomatic aneurysm, size more than 2.5 cm, rapid increase in size with impending rupture [2].

A transplant nephrectomy is deemed to be the definitive treatment for post renal transplant aneurysm [11]. In cases where the graft can be preserved, endovascular stenting, percutaneous thrombin injection or open surgical repair can be considered.

Open surgical options for TRAA includes transplant nephrectomy, creation of vascular anastomoses [11], as well as traditional surgical methods like aneurysmectomy which is the most commonly described surgical method for the repair of renal artery aneurysm [12]. Aneurysectomy has not been described as a treatment for TRAA. Vascular anastomoses can be created through various such as a saphenous vein graft, autogenous internal iliac artery bypass, third party vascular allografts, or even Polytetraflouroethylene (PTFE) Graft. In select cases of variation of anatomy or an atheromatous external iliac artery, the internal iliac artery can be considered as a alternative blood supply to the transplanted kidney. However, the internal iliac artery is less commonly used due to the risk of compromising distal vascular supply to the pelvis, resulting in impotence, sexual dysfunction and buttock claudication [13]. Complications relating to using the hypogastric artery may be reduced by opting for this in females as opposed to males, and performing a side to end anastomosis rather than an end to end anastomosis, as described by IH Mohamed et al. [13]. Third party vascular allografts are most commonly used for living donor kidney transplants for ex vivo reconstruction of the vessels of the allograft. Complications include developing anti HLA antibodies against the graft [14]. PTFE grafts are prosthetic vascular grafts that may be used as a substitute to organic grafts. They are advantageous due to their unlimited supply compared to autogenous vascular grafts. While the short-term patency rates of PTFE grafts are comparable to that of organic grafts, the long term patency rates remains unclear. The complications of PTFE grafts include that of adhesions, migration, thrombosis and infection [15].

In deciding the approach and treatment to this case, we considered endovascular stenting, percutaneous thrombin injection and surgical repair. As discussed above, an open surgical approach was deemed to be the most feasible.

Although not commonly done, we demonstrate that a saphenous vein graft can be used as a conduit when endovascular and percutaneous approaches are not feasible.

## Conclusion

TRAA, albeit rare, is a complication that can occur post renal transplant. Surgical repair of TRAA is feasible with a multidisciplinary approach and careful preoperative planning. Saphenous vein graft is still a versatile graft and can be used as a conduit successfully and a better outcome can be achieved.

## Data Availability

The datasets used and/or analysed during the current study available from the corresponding author on reasonable request.

## Abbreviations

TRAA: Transplant Renal Artery Aneurysm
CTA: Computed Tomography Angiogram
ESRD: End Stage Renal Disease
LDKT: Living Donor Kidney Transplant
eGFR: Estimated Glomerular Filtration Rate
CIA: Common Iliac Artery
EIS: External Iliac Artery
PTFE: Polytetraflouroethylene
